# Sudden cardiac death owing to arrhythmogenic right ventricular cardiomyopathy: Two case reports and systematic literature review

**DOI:** 10.1097/MD.0000000000008808

**Published:** 2017-11-27

**Authors:** Jiao Mu, Guohui Zhang, Dazhong Xue, Mengrou Xi, Jiarui Qi, Hongmei Dong

**Affiliations:** a Department of Forensic Medicine, Hebei North University, Zhangjiakou, Hebei; b Department of Forensic Medicine, Tongji Medical College of Huazhong University of Science and Technology, Wuhan, Hubei, PR China.

**Keywords:** arrhythmogenic right ventricular cardiomyopathy, autopsy, sudden cardiac death

## Abstract

**Background and objective:**

Arrhythmogenic right ventricular cardiomyopathy (ARVC) is increasingly recognized in forensic practice with controversial diagnosis. Here we described the epidemiological characteristics and reported the pathogenetic mechanism, diagnostic challenges, and forensic implications of Chinese ARVC autopsy cases.

**Methods:**

Two cases of sudden cardiac death owing to ARVC were reported. Retrospective analysis were performed on such 2 cases and 45 cases of separate ARVC complete autopsy case reports through Chinese literature databases in the last 30 years.

**Results:**

There were 27 males and 20 females, and the mean age at death was 35 years. Sudden cardiac death was the first manifestation observed in most patients, with no previous family and medical history. Exercise, acute stress, increased cardiac workload, and ethanol are frequently involved. The mean heart weight was 393 g (range, 240–590 g), and 10 cases had relative heart hypertrophy. Microscopic abnormalities included replacement of myocardium by adipose infiltration in 68.09% cases and fibroadipose in 31.91% cases; 80.85% cases were restricted to the right ventricle (RV), whereas biventricular subtype was seen in the remaining 19.15% cases. The preliminary quantitative histology showed 60.7% of fat tissues, 12.1% of fibrosis, and 27.2% residual myocytes in RV. Inflammatory cell infiltration was found in 25.53% cases, but myocyte necrosis was found in only 1 case. In 10.64% of cases, cardiac conduction was infiltrated by fibrosis, adipose, or both.

**Conclusion:**

In this review, the most characteristic and distinct histopathologic features that are diagnostic or highly suggestive of ARVC for forensic pathologists were identified. Combining gross and histological examinations with postmortem genetic analysis is recommended for identifying ARVC.

## 1 Introduction

Sudden cardiac death (SCD) is one of the most important causes of death worldwide. SCD accounts for an estimated 450,000 deaths, or 15% of total annual deaths in the United States.^[1]^ In China, the incidence of SCD was 41.8/100,000, and about 544,000 people die from SCD every year.^[2]^ Thus, diagnosis of SCD remains a major challenge in forensic medicine worldwide. In recent years, arrhythmogenic right ventricular cardiomyopathy (ARVC), which is a relatively rare but probably an underestimated cause of SCD, has received widespread attention from the medicolegal community.

ARVC is poorly understood and often underdiagnosed disorder of the right ventricle (RV) at postmortem, characterized by replacement of myocardium by fibroadipose tissue. The prevalence of ARVC was estimated to be 1 in 5000 people and accounted for up to 20% of SCDs in people <35 years of age.^[3,4]^ In a series of 86 sudden death cases as reported by Zhao et al,^[5]^ ARVC accounted for 10.3% of all SCD cases and remained as the second major cause of SCD.

The definitive diagnosis of ARVC is based on the known electrophysiological, structural, histological, and familial characteristics in clinical practice.^[6]^ However, sudden death is often the first manifestation in ARVC patients and forensic pathologists often encounter these cases without any history of clinical symptoms and familial characteristics.^[7]^ Moreover, there are no universally accepted autopsy criteria for diagnosing ARVC. So, accurate diagnosis of ARVC remains a challenge for forensic pathologists.

Very few sporadic autopsy cases have been reported in China. Hence, to clearly understand SCD owing to ARVC in Chinese patients, our study retrieved and described the epidemiological characteristics of 47 Chinese ARVC autopsy cases. The pathogenetic mechanism, diagnosis challenges, and forensic implications of ARVC are also discussed.

## 2 Methods

### 2.1 Study sample

Two autopsy cases were acquired by Tongji Forensic Medical Center and Hebei Northern Forensic Medical Center. The causes of death in all cases were determined after complete and systematic autopsy and toxicological analysis. Medical and family history, case information, macroscopic and microscopic findings were reviewed in all the cases.

Between January 1986 and September 2017, 45 Chinese ARVC autopsy cases were retrieved using “Arrhythmogenic right ventricular dysplasia/cardiomyopathy (ARVC/D) and autopsy and forensic” as the free word or keyword from an electronic search of CNKI, WAN FANG, WEI PU, SINOMED, DU XIU, CHAO XING, Baidu, Mediline, PubMed, Cochiane Library, and Web of SCI databases. The references cited in the retrieved articles were also examined to identify for additional reports.^[8–25]^

### 2.2 Macroscopic and microscopic examinations

In 2 autopsy cases, the cardiac tissue samples were taken from the affected anterior walls of the right ventricles. The slides were stained with both hematoxylin & eosin (H&E) and Masson trichrome to differentiate between fibrous and cardiac tissues. A slide scanner Zeiss Axio Scan Z1 (Carlzeiss Macroscopy GmbH, Germany) was used to scan the slides. The slides stained with Masson trichrome were scanned by ×40 at 100%. Image-Pro-Plus Version 6.0 was used to calculate the areas of the heart muscle, fibrous, and fatty tissue.

### 2.3 Ethical statements

The research protocols regarding the autopsy cases were approved by the ethics committee of Hebei North University and Huazhong University of Science and Technology. Informed consent was obtained from each claimed case.

### 2.4 Statistical analysis

Statistical analyses were conducted using SPSS 20.0 (SPSS Inc, hicago,IL). Continuous data are presented as mean ± standard deviation, whereas categorical variables were expressed as number and/or percentage.

## 3 Results

### 3.1 Age and sex distribution

A total of 47 ARVC autopsy cases were identified between 1986 and 2017. There were 27 males (57.45%) and 20 females (42.55%), with an age range of 13 to 57 years. Male to female incidence ratio was 1.35:1. The mean age at death was 35.35 ± 11.34 years, and >75% of individuals were in the age range of 15 to 45 years.

### 3.2 Family and clinical history

SCD owing to ARVC demonstrated unknown antemortem diagnosis, with no previous family and medical history in all the cases. Sudden death was the first manifestation observed in most of the ARVC cases. A 30-year-old man had a cardiac disorder with palpitations, whereas another 23-year-old man had a history of syncopal episodes before death. A 42-year-old woman had symptoms of right cardiac dysfunction, which included abdominal distention, jugular venous distention, hepatomegaly, and lower limb dropsy. A 26-year-old man and a 37-year-old woman showed abnormal electrocardiograms of ventricular fibrillation during resuscitation.

### 3.3 Situations before and near death

Detailed records of the situations before and near death in 27 cases were described. Two persons died at rest, 3 at work, and 5 in sleep. Seven persons experienced a stressful situation owing to quarrel (2 cases), minor injury (2 cases), childbirth (1 case), detention (1 case), and son's sudden death (1 case). Seven persons died because of sudden increase in the cardiac workload: venous transfusion (4 cases), competitive sports (2 cases), and morning exercise (1 case). In addition, 3 persons died after drinking alcohol.

### 3.4 Macroscopic findings

Detailed records of the weight of the heart in 21 cases were recorded. The mean heart weight was 393.13 ± 50.37 g (range, 240–590 g), 398.57 ± 76.69 g in males (range, 310–520 g), and 388.18 ± 75.43 g in females (range, 240–590 g). Ten of 21 cases had relative heart hypertrophy, whereas 16 cases had chamber enlargement. The left ventricular (LV) thickness ranged from 10 to 16 mm (13.6 ± 1.5 mm), and 5 cases had LV hypertrophy (≥15 mm). The RV thickness ranged from 1 to 10 mm (4.1 ± 0.8 mm), and the interventricular septal (IVS) thickness ranged from 8 to 13 mm (11.2 ± 1.4 mm). Mild coronary artery stenosis was observed in 3 cases and RV aneurysm in 1 case. Visual inspection revealed prominent fatty infiltration in the RV myocardium in 37 cases and LV myocardium in 3 cases. Other cases showed no remarkable changes on gross examination.

### 3.5 Microscopic findings

Microscopic examination showed myocardial replacement by diffuse and segmental fatty or fibrofatty tissue, and the residual degenerative myocytes were scattered as islands and fragments. The fatty or fibrofatty tissue was restricted to RV in 38 cases (80.85%), and was biventricular in the remaining 9 cases (19.15%). Fatty pattern was observed in 32 cases (68.09%), and fibrofatty pattern was observed in 15 cases (31.91%). Transmural RV fatty infiltration was observed in 18% of patients. Inflammatory infiltrates were present in 25.53% of the cases, but myocyte necrosis was found in only 1 case. In 10.64% of cases, the cardiac conduction was infiltrated by fibrosis, adipose, or both.

### 3.6 Quantification of fibrosis, fat, and muscle tissue in ARVC

The fibrosis, fat, and muscle tissue were not quantified in most of the cases from literature. In 2 autopsy ARVC cases from our institution, we attempted to quantify the fibrosis, fat, and muscle tissue in RV. Results showed 60.7% ± 15.1% of fat tissue, 12.1% ± 9.4% of fibrosis, and 27.2% ± 13.7% residual myocytes in RV.

## 4 Discussion

ARVC, also called right ventricular dysplasia (RVD) and right ventricular cardiomyopathy (RVC), was uniformly defined and classified as 1 of the 5 primary cardiomyopathies in 1995. ARVC occurs in both the sexes at any age, but sudden deaths tend to occur in adults between 15 and 45 years, with a mean age of about 30 years.^[26,27]^ The male predisposition might be associated with the disease genes and androgen hormone. Tabib et al^[26]^ examined 200 cases of sudden death owing to ARVC and found that the mean age was 36 years (range, 5–64 years), and 108 (54%) cases of these were males. Our study revealed similar age range of 13 to 57 years (mean 35 years), which was slightly a male-dominant ARVC cohort, and >75% of the deaths occurred in patients between 15 and 45 years.

Clinical presentation of ARVC typically involves palpitations, syncope, ventricular tachycardia, congestive heart failure, and SCD.^[28,29]^ In the present study, only 3 patients had a history of syncope, ventricular tachycardia, or right cardiac dysfunction. As sudden death is the first sign observed in most of the ARVC cases, no further medical examinations or history can be provided. Lack of clinical history frames a very challenging diagnosis for forensic pathologists.

Previous studies have indicated that strenuous activity was closely related to sudden death in ARVC. Studies from Spain and France demonstrated that half of the ARVC patients died during exercise.^[30,31]^ Our data showed that under several stressful conditions in daily life, other conditions such as quarrel, minor injury, childbirth, and mood are also frequently involved. During stressful situations, increased catecholamine release and parasympathetic stimulation lead to lethal arrhythmia.^[32]^ In addition, alcohol is another notable factor involved in the death of ARVC patients. Cittadini et al^[33]^ reported 1 case of SCD owing to synergic effect of cocaine and ethanol in an ARVC patient. Ethanol can increase myocardial oxygen demand and cause irregular heart rhythms. These subsequently exacerbate the ventricular instability of preexisting cardiac substrate and increase the risk of cardiotoxicity.^[33]^

The exact pathogenesis of ARVC is still unclear, but this involves a genetic factor.^[34]^ Currently, the known genetic mutations associated with ARVC include PG, PKP2, DSP, DSC2, DSG2, TGFβ3, TMEM43, RYR2, TTN, and JUP.^[35]^ Most of the patients reported had a family history and genetic tendency.^[36]^ However, the patients included in this study had no family history of ARVC or SCD, and this may be associated with the absence of comprehensive clinical data. Currently, molecular and genetic testing of ARVC are not a routine diagnostic procedures. However, genetic testing is recommended to be a useful in dealing with the suspected ARVC cases at autopsy and consequently identifies the cardiac risk of living family members.

Heart weight in ARVC cases was within the normal range, but was increased by varying degrees in most cases. In the study by Basso et al,^[37]^ the heart weighed between 270 and 600 g, with an average weight of about 400 g. Our study revealed an average heart weight of 393 g. Moreover, 21% of ARVC cases in our study had relative heart hypertrophy. The primary feature of ARVC is focal or diffuse replacement of ventricular myocardium by adipose tissue.^[37]^ Whereas triangle of dysplasia of the RV is most frequently involved, and at times the LV is affected in some cases.^[38]^ Our results showed that ARVC also affected both the ventricles in 19% of cases, and so, the recently proposed nomenclature of “arrhythmogenic cardiomyopathy” (ACM) was considered to be more reasonable. Indeed, large areas of fat infiltration could be observed on gross examination, but 19% of medicolegal cases in the present study lacked obvious macroscopic fat infiltration. Definitive diagnosis needs an adequate sampling of myocardial tissue and careful histological examination. This disease may often be overlooked if the forensic pathologist is unaware of its existence. Thus, in suspected SCD cases, the heart must be deliberately sectioned and extensively sampled for histological analysis to check for the presence of ARVC.

Currently, 2 microscopic patterns are known, fatty or fibrofatty.^[28]^ These might represent 2 consecutive stages of cardiomyopathy.^[39]^ The fatty type was observed to be as high as 68% in our cases, and was believed to have a higher risk for sudden death. However, it still remains controversial whether pure fatty infiltration of the RV is considered a morphologic hallmark of ARVC. In fact, a certain amount of fatty infiltration is present in fatty heart and 50% of normal hearts in the elderly.^[40]^ Simultaneously, some researchers demonstrated that the pathological pure fatty infiltration may also be a phenotype of ARVC as ARVC-related genetic mutations could be identified in these kind of cases.^[35]^ Thus, when dealing with a case of sudden death, where the only morphologic finding is an increased amount of epicardial or intramyocardial fat, it is difficult for forensic pathologists to make an exact diagnosis.

According to our view, 2 key points should be noted regarding the morphological diagnosis of ARVC. The first one is to identify the proportion of adipose replacement and isolation of myocytes by adipose. As stated by a morphological quantitative study,^[41]^ the proportion of fat tissue in the RV was >80% in ARVC patients. Chen et al^[25]^ collected 8 autopsy cases of sudden death because of ARVC and found 68.3% of fat tissue in the RV and 23.8% in the residual myocytes within the areas of fatty infiltration, and these findings were similar to our study results. Currently, the new clinical universal criteria demonstrated specific quantization standard and clarified that the residual myocytes should be <60% (or is estimated to be at <50%) to exclude the cases with slight or moderate lipomatosis in RV.

Another key point to note is that ARVC is not just a matter of fat. Fatty heart, also called fatty infiltration or fatty ingrowth pathologically, is present as continuous fat infiltration in the biventricular epicardium. Unlike ARVC, fatty heart usually occurs in obesity, coronary artery disease, and women of advanced age.^[42,43]^ Histologically, fatty heart shows a pure fatty replacement and the normal fat locality is usually limited to the intramural arteries and nerves.^[43]^ Generally, the boundary between the inner myocardium and the outer subepicardial fat is relatively distinct. However, in ARVC, the fats penetrate into the myocardium without obvious demarcation.^[42]^ Moreover, 2 important histologic features are important to provide a definitive diagnosis of ARVC, which include significant fibrosis or degenerative changes of the myocytes entrapped within the fibrous/fatty tissue. Myocardial inflammatory infiltrates, fibrosis, anomalous pathways or necrosis should be searched for a more convincing diagnosis.^[42]^ Demellawy et al^[43]^ further put forwarded the following features, such as RV or biventricular cavities dilatation, RV wall thinning, aneurysm formation and multiple, but the subtle foci of myocarditis are included as diagnostic or suggestive pathological criteria.

Currently, there are no consistent autopsy criteria to definitively diagnose ARVC. Based on the literature review and the present anatomic study, we proposed the most important characteristic and distinct histopathologic features that are diagnostic or highly suggestive of ARVC (Table 1) in forensic practice. These may be helpful for forensic pathologists to make a reliable diagnosis even in the absence of a clinical history. The strict unified autopsy diagnosis of ARVC is still imperative.

**Table 1:**
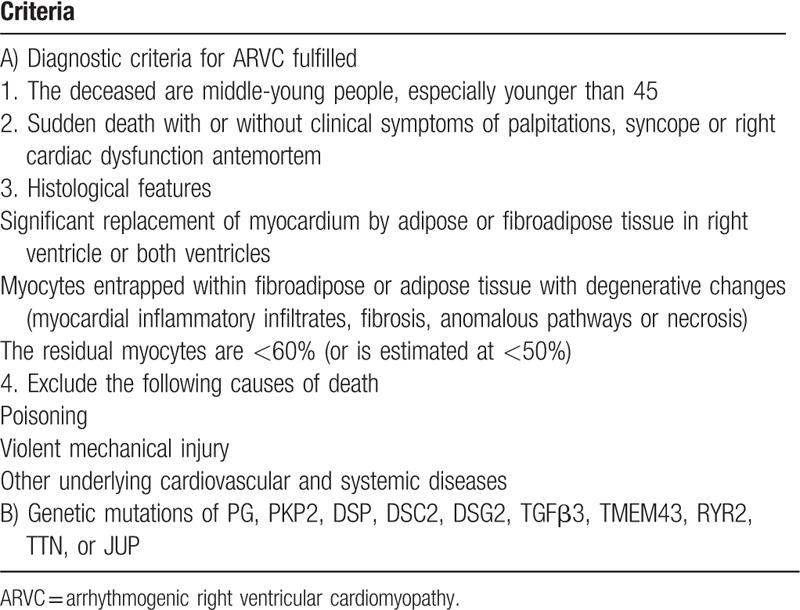
Possible diagnostic criteria or highly suggestive of ARVC in forensic pathology.

## 5 Conclusion

Our study findings demonstrated that ARVC usually occurred in younger people, and sudden death is often the first manifestation seen. Exercise, acute stress, increased cardiac workload, and ethanol are frequently involved in the occurrence of ARVC. Most of the ARVC patients had no clinical data or family background, and the typical pathological changes may not be grossly observed during autopsy. When dealing with cases of sudden death, where the only morphologic finding was adipose infiltration, forensic pathologists should extensively search for more convincing arrhythmogenic pathological findings, such as myocardial inflammatory infiltrates, fibrosis, anomalous pathways, and necrosis. In the suspected cases, postmortem genetic testing might be helpful for producing diagnostic accuracy.

## 6 Limitations

Our study does not completely reflect the epidemiological characteristics of ARVC autopsy cases in China. Some autopsy ARVC cases were not collected as autopsies were not performed or might be misdiagnosed. Furthermore, some ARVC autopsies might not be reported.
